# Garlic supplementation attenuates cycling exercise-induced oxidative inflammation but fails to improve time trial performance in healthy adults

**DOI:** 10.1080/15502783.2023.2206809

**Published:** 2023-05-12

**Authors:** Jung-Piao Tsao, Jeffrey R. Bernard, Tse-Hsin Tu, Hsiu-Chen Hsu, Chia-Chen Chang, Su-Fen Liao, I-Shiung Cheng

**Affiliations:** a China Medical University, Department of Sports Medicine, Taichung, Taiwan; b California State University, Department of Kinesiology and Public Health Promotion, Turlock, CA, USA; c National Taichung University of Education, Department of Physical Education, Taichung, Taiwan; d Central Taiwan University of Science and Technology, Physical Education Office, Taichung, Taiwan; e National Dong Hwa University, Center for Physical Education, College of Huilan, Hualien, Taiwan; f Changhua Christian Hospital, Department of Physical Medicine and Rehabilitation, Hualien, Taiwan; g National Chung Hsing University, Department of Post-Baccalaureate Medicine, College of Medicine, Taichung, Taiwan

**Keywords:** Ergogenic supplement, exercise performance, fatigue

## Abstract

**BACKGROUND:**

Garlic extract has been shown to enhance antioxidant and anti-inflammation activities in humans. The present study investigated the effects of garlic supplementation on 40-km cycling time trial performance, exercise-induced oxidative stress, and inflammatory responses in healthy adults.

**METHODS:**

Eleven healthy males were recruited to perform this single-blind crossover study. Participants were randomly assigned to either garlic (garlic extracts 1000 mg/d for 4 weeks) or placebo trials. Following 4-wks of supplementation, participants performed a 40-km cycling challenge. Total cycling performance time and respiratory exchange ratio (RER) were recorded. Blood samples were collected every 10 km to determine exercise-induced oxidative stress, inflammation, and muscle damage.

**RESULTS:**

The 40-km cycling time trial performance was not improved following 4 weeks of garlic supplementation. However, 4-wk garlic supplementation significantly increased whole-body antioxidant capacity (total antioxidant capacity, TAC), and subsequently attenuated MDA, TNF-α, and LDH during the 40-km cycling exercise period (*p* < 0.05). There were no significant differences among the blood biomarkers glucose, NEFA, IL-6, UA, and CK respectively. The respiratory exchange ratio was similar between garlic and placebo trials.

**CONCLUSION:**

Four-week oral garlic supplementation attenuates exercise-induced oxidative inflammation and muscle damage during a 40-km bout of cycling. However, it appeared that 4-wk oral garlic had no ergogenic effect on cycling performance in healthy males.

## 1. Introduction

During high-intensity exercise, the mitochondrial electron transport chain of muscle cells generates vast amounts of reactive oxygen species (ROS) and free radicals [1]. Exercise-induced production of free radicals exceeds the ability of the whole-body antioxidant defense system, thus, disrupting redox balance [2]. At the same time, elevated concentrations of malondialdehyde (MDA), interleukin-6 (IL-6), and tumor necrosis factor-α (TNF-α) are important blood indicators of systemic oxidative stress and inflammation [3]. As oxygen consumption increases, oxidative stress, inflammation, and damage to the structure of muscle cells occur. Blood markers used to indicate muscle damage, such as creatine kinase (CK), lactate dehydrogenase (LDH), and uric acid (UA) levels [4–6] all increase in response to exercise. Muscle tissue damage caused by high-intensity and/or prolonged exercise relates to ROS-induced macrophage infiltration and inflammation levels. This phenomenon is likely due to increased oxidation levels in cells and tissues, which damages the cell membrane resulting in tissue inflammation and damage [7,8]. Therefore, improving systemic antioxidant capacity during exercise is an important key factor in preventing muscle damage induced by high-intensity exercise. At this time, research on flavonoid-health foods, such as quercetin and resveratrol, have been shown to increase total antioxidant capacity (TAC), as well as reduce oxidative stress, levels of inflammation, and levels of muscle damage [9,10]. Collectively, these factors work to enhance exercise performance. However, the physiological effects of garlic supplementation on oxidative stress MDA, inflammatory levels IL-6, TNF-α, muscle damage CK, LDH, and UA, all of which negatively affect exercise performance, have not been thoroughly explored. Furthermore, it is not known how garlic supplementation impacts overall exercise performance.

Garlic is comprised of phytochemicals such as flavonoids, sulfur-containing compounds (S-allylcysteine, SAC), allicin (allyl 2-propenethiosulfinate or diallyl thiosulfinate), ajoene, etc. Both animal and human studies suggest that oral garlic supplementation could contribute to reduced whole-body oxidative stress and inflammation [11,12]. Furthermore, SAC provides protective effects against cellular damage caused by hydrogen peroxide [13], and alliin can not only scavenge free radicals [14] but also increase glutathione concentration to further improve antioxidant capacity [15]. Studies on the effects of garlic extract on exercise in humans have been limited. Su et al. reported that 14 days of garlic supplementation (800 mg/day) significantly reduced any cellular damage and inflammation caused by eccentric exercise (downhill running). It was also found that garlic supplementation improved antioxidant capacity and assuaged the muscle damage induced by eccentric exercise [16]. Additionally, an investigation conducted by Gholami et al. (2006) showed that when 30 minutes of intense exercise was proceeded by an acute dose of garlic supplementation (1000 mg), the amount of cellular damage was unaffected, whereas exercise-induced lipid peroxidation MDA concentration was improved [17]. Although, both acute and short-term garlic supplementation appears to mitigate the adverse physiological reactions caused by exercise, specifically as it relates to oxidative inflammation and muscle damage. However, there are currently no human studies showing evidence that garlic supplement improves cycling performance under attenuating exercise-induced oxidative stress.

Athletes prefer to consume antioxidants in the form of whole foods or supplements in order to relieve exercise-induced oxidative stress, inflammation, and muscle damage. Physiologically, garlic extracts prevent increased inflammation levels of TNF-α, IL-6, and c-reactive protein (CRP) by increasing the release of hydrogen sulfide. This is most likely due to enhanced antioxidant endogenous products [11,12], in addition to reducing intracellular oxidative stress, slowing down TNF-α response, and inhibiting activation of nuclear factor kappa *B* (NF-κB) inflammatory pathway [18]. Moosavian et al. showed that in rheumatoid patients treated with garlic supplement (500 mg/d for 8 weeks) or placebo, TAC levels were significantly increased, and MDA levels were significantly decreased by garlic supplementation [19]. A systematic review and meta-analysis by Moosavian et al. also revealed that treatment of humans with garlic supplementation modulated the dose-effect of oxidative stress-related markers (dose >1000 mg/day, intervention time<8 weeks), including ergogenic properties such as increased systemic TAC and decreased MDA levels [20]. Nevertheless, the efficacy of garlic supplementation for different durations and doses on oxidative stress and antioxidant capacity requires more human studies in order to validate its usage as a supplement. Therefore, we sought to determine the effects of garlic supplementation in exercising male participants. In present human study, we measured time to exhaustion to a 40-km cycling challenge, the blood energy biomarkers glucose and nonesterified fatty acids (NEFA), as well as oxidative stress (TAC and MDA) inflammatory markers (IL-6 and TNF-α), and muscle injury (CK, LDH, and UA). We hypothesized that daily garlic supplementation (1000 mg/day for 4 weeks) would improve cycling performance by relieving systematic oxidative stress, inflammation, and muscle injury.

## 2. Materials and methods

### 2.1. Participants

Eleven physically active males were recruited to perform this single-blind crossover design study. G*Power（3.1.9.4）software was used to calculate sample size as previously detailed [10]. An effect size of 1.15 was found in the present study while the statistical power was set at 90%. Prior to beginning the study, all participants completed a medical health questionnaire. Those that exhibited a history of cardiovascular disease, diabetes, musculoskeletal and/or neuromuscular problems were excluded from the study. Anyone that was unable to complete the $\dot{\;}V$O2max test was also excluded. The present experimental protocol was reviewed and approved by the Institutional Review Board at the University of Taipei, in Taiwan (IRB-2019-074). Moreover, participants received complete instructions and signed the consent form. Participants were allowed to voluntarily excuse themselves from the study for no reason.

### 2.2. Experimental design and procedure

This investigation utilized a single-blind crossover study design. Participants were randomly assigned to either garlic supplementation (1000 mg, GNC Holdings Inc., USA) or a placebo group. Supplementation was administered over a 4-wk period. The capsules provided for both the garlic and placebo treatment groups were identical in appearance, thus the participants were unaware which treatment they received. After the 28-d washout period, all subjects are changed to perform the different treatments on either garlic or placebo supplementation. Participants performed a standardized $\dot{\;}V$O2max test 7 days prior to the exercise challenge on a fixed cycle ergometer (Monark Exercise, Varberg, Sweden) while wearing a gas analyzer (Cortex Biophysik, Nonnenstrasse, Leipzig, Germany) [21], and the value was used to convert to the exercise intensity of 50% $\dot{\;}V$O2max for the 40-km cycling time trial in the present study. All participants were asked to not make any drastic changes to their normal routines, such as to maintain their regular exercise and dietary regimes throughout the duration of the study. They were also asked to not smoke, consume caffeine, and to not take drugs for anti-oxidative stress and/or inflammatory until the end of the study. Participants received a standard meal (lunch/dinner: total energy 700.60 kcal per meal, carbohydrate: 77.90 g; Fat: 26.60 g; Protein: 37.10 g) approximately 24 hours before the exercise challenge for both trials. On the day of the experiment, participants reported to the lab at 7 a.m. to establish baseline (B) values for body composition, gas, and blood markers for baseline. After such measurements were completed, they were allowed to consume their standardized breakfast. One hour after breakfast, all participants performed the 40-km cycling challenge at 50% $\dot{\;}V$O2max intensity at their preferred cadence (measured in revolutions per minute, rpm), and were required to complete 30-sec sprints every 3 km and for 1 km at the end of the 39 km [22]. Time was recorded when finishing the cycling time trial. Meanwhile, blood and gas samples were collected as detailed below.

### 2.3. Blood sample collection and analysis

All venous blood collections were performed by a qualified medical technologist during the blood collection procedures. The samples were centrifuged at 3000 rpm (1006.2 g) for 10 minutes under 4°C. Plasma was collected in the supernatant and frozen at −80°C for later analysis.

Blood samples were collected at the 10, 20, 30, and 40 km marks during the exercise challenge. Blood glucose was measured using an automated glucose analyzer (YSI Life Sciences, Yellow Springs, OH, USA). Using an automatic photometric analyzer (Hitachi 7020, Japan), commercially available kits were applied to determine the plasma concentrations of NEFA (WAKO, Germany), CK (Cayman Chemical Company, ANN Arbor, MI, USA), LDH, and UA (Kanto Chemical, Kanagawa, Japan). For TAC and MDA, absorbance was measured at wavelengths of 734 and 534 nm, respectively, using the UV-visible spectrophotometer (Tecan GENios, A-5082, Austria). Similarly, the absorbance of cytokine IL-6 and serum TNF-α was read at 450 and 570 nm wavelengths to calculate concentrations in the serum samples. BioLegend reagents (Bio-Legend Inc, San Diego, CA) were used for the spectrophotometer measurements. A more detailed account of the evaluation for energy metabolism, oxidative stress, inflammation, and muscle damage can be found in our previously published studies [9,10].

### 2.4. Gas collection and analysis

Gas analyzers (Cortex Biophysik, Nonnenstrasse, Leipzig, Germany) were used to collect gas samples from participants every 10 km throughout the 40-km cycling challenge (i.e. 10, 20, 30, and 40 km). Gas analysis was used to assess RER values to determine the systematic metabolic trend. RER was based on the ratio of CO_2_ to VO_2_ (CO2/O2). In addition, the oxidation rates of carbohydrates and fat were calculated using the equation by Frany [23].

### 2.5. Statistical analyses

All values were expressed as mean ± standard error (mean ± SE). Statistical analysis was performed using SPSS software. A paired t-test was used to analyze the time recorded by participants during the 40 km time trial. Data for blood and gas samples collected at different time points were analyzed by repeated measure two-way ANOVA (trial × time). If a significant interaction was observed between treatments and time points, we further conducted a simple main effects analysis. In this regard, for post hoc analysis, Fisher’s least significant difference (LSD) was used. The α value was set at *p* < 0.05.

## 3. Results

### 3.1. Garlic extract did not improve 40-km cycling time trial performance and had no effect on whole-body energy metabolism during exercising

**Figure 1.**
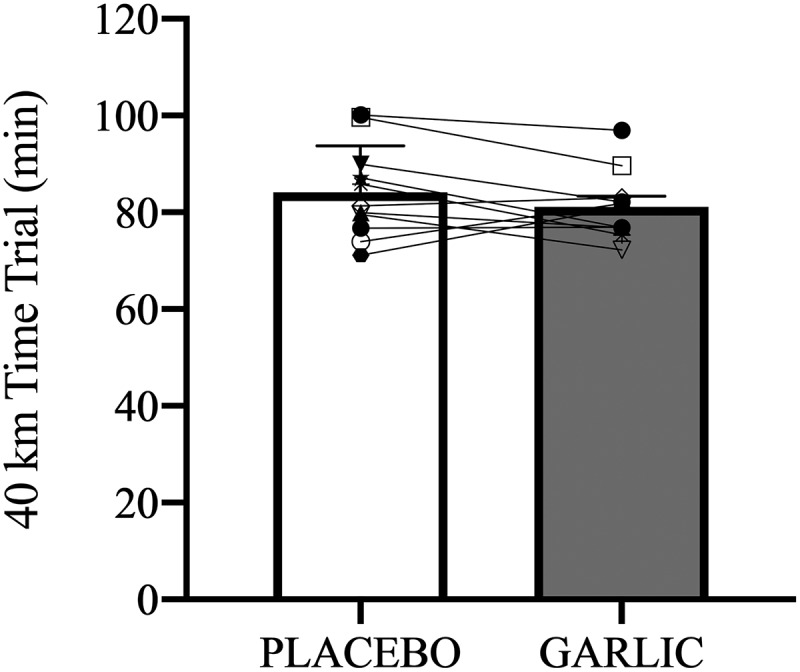
Time trial of individual data and mean values data of all participants on the 40-km cycling performance between the garlic and placebo. Values are expressed as mean ± SE, *N* = 11.

**Figure 2.**
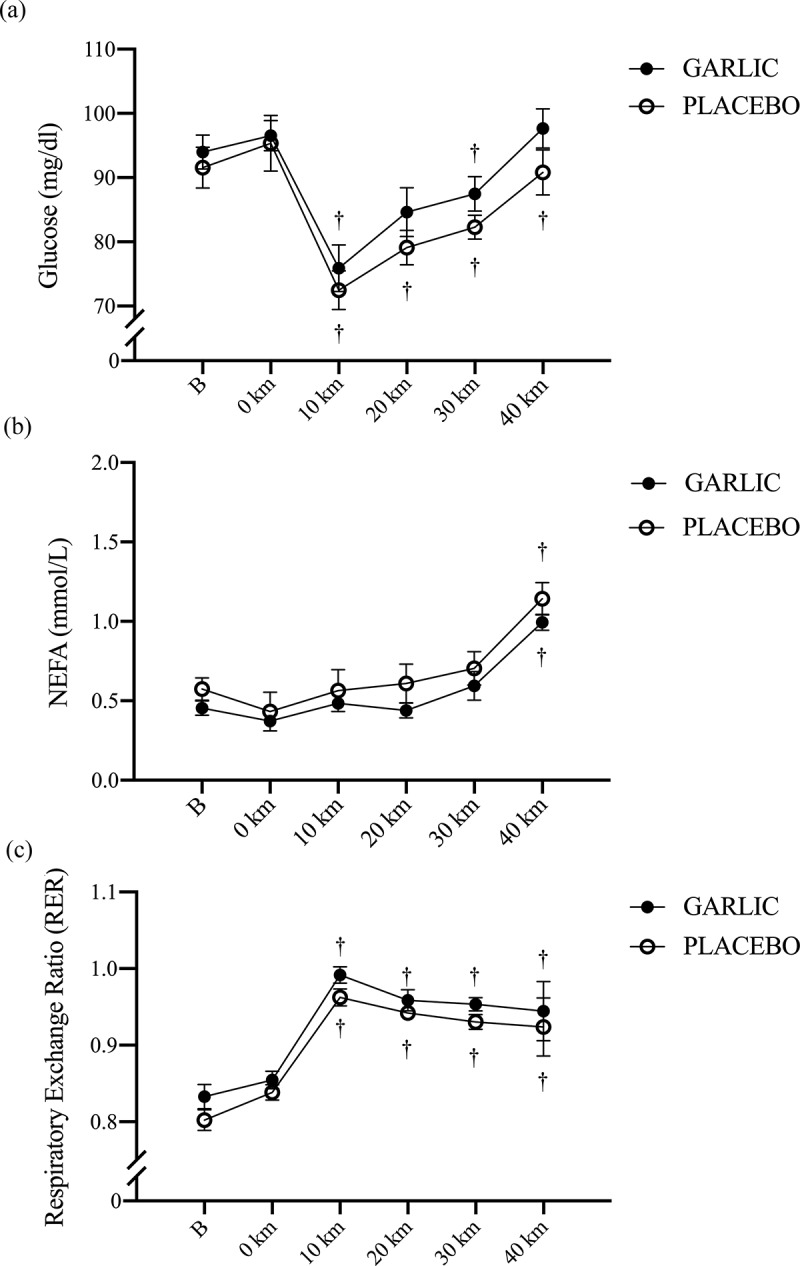
Blood glucose (a), plasma non-esterified fatty acids (NEFA) (b), and respiratory exchange rate (c) in (-●-) garlic and (-○-) placebo trials. B: represents before the 40-km cycling time trial. + Significant difference against B point in the same trial. (*p* < 0.05). Values are expressed as mean ± SE, *N* = 11.

Table 1 shows the physiological characteristics for all subjects in the present study. Following 4-wks of oral supplementation, there was no significant improvement in the 40-km cycling time trial performance after garlic supplementation compared to placebo trials (garlic trial: 81.18 ± 2.12 (min); placebo trial: 84.14 ± 2.88 (min), *p* > 0.05, figure 1). Identically, no significant differences were observed for plasma glucose (Figure 2(a)) free fatty acids (Figure 2(b)), and respiratory exchange ratios (Figure 2(c)) between the two trials. Calculating the area under the curve (AUCs) did not reveal any differences between trials for carbohydrate oxidation (garlic trial: 71.84 ± 6.67 (g/min); placebo trial: 64.85 ± 5.38 (g/min), *P* > 0.05) and fat oxidation (garlic trial: 5.50 ± 2.01 (g/min); placebo trial: 8.18 ± 1.54 (g/min), *P* > 0.05). Therefore, it does not appear that garlic supplementation has an impact on substrate utilization, and specifically on whole-body fat oxidation while performing a 40-km cycling exercise challenge.

**Table 1.** Participant characteristics.

Variables	Value
Age (year)	22.0 ± 4.8
Height (cm)	174.9 ± 7.7
Weight (kg)	77.0 ± 13.3
Body fat (%)	17.4 ± 5.3
Body mass index (kg/m2)	24.04 ± 4.14
Maximum oxygen consumption (ml/kg/min)	45.3 ± 5.5

Data are expressed as mean ± standard deviation (SD).

### 3.2. Garlic extract attenuated mediators of oxidative stress and inflammation during exercise

**Figure 3.**
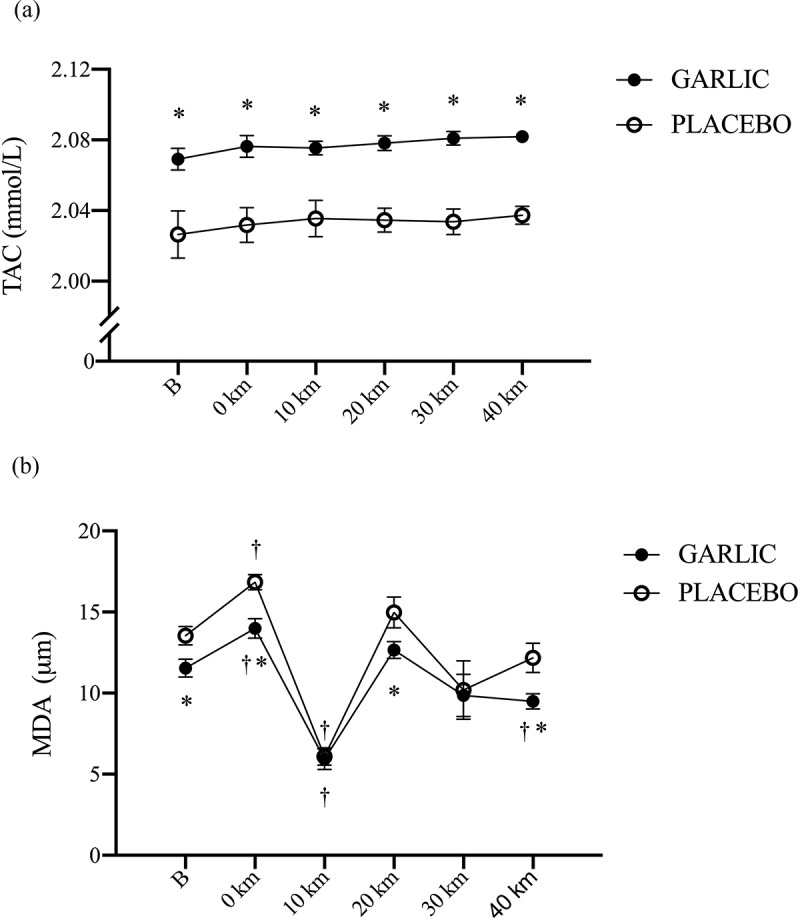
Total antioxidant capacity (TAC) (a), and malondialdehyde (MDA) (b) concentrations in (-●-) garlic and (-○-) placebo trials. B: represents before the 40-km cycling time trial. * Significant difference between garlic and placebo (*p* < 0.05). + Significant difference against B point in the same trial. (*p* < 0.05). Values are expressed as mean ± SE, *N* = 11.

**Figure 4.**
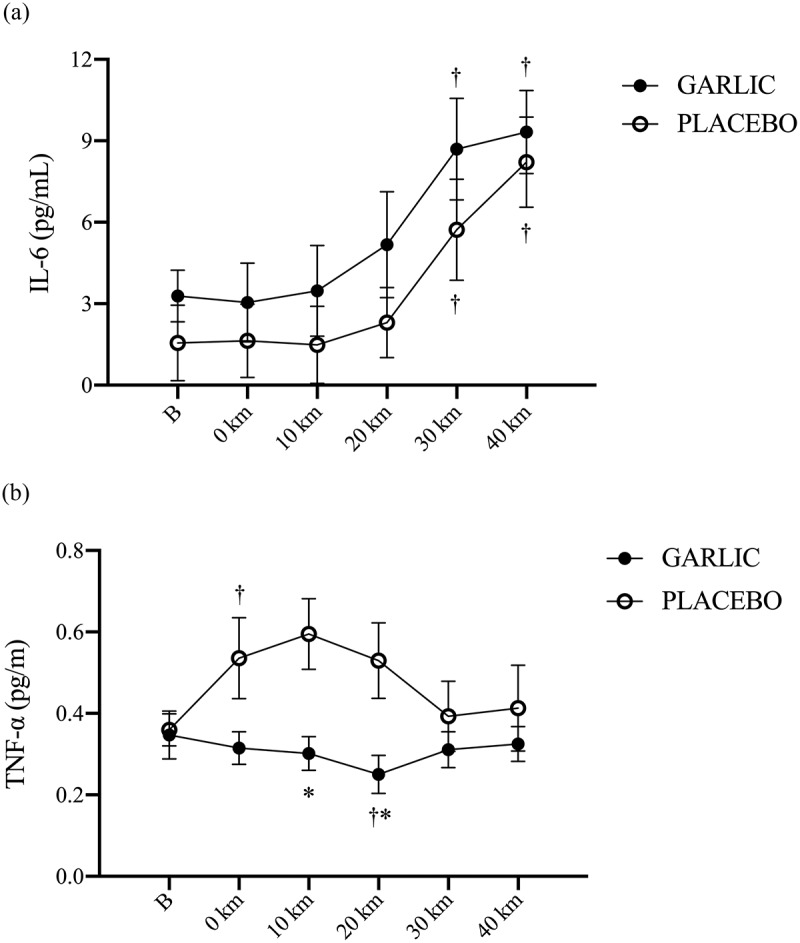
Interleukin-6 (IL-6) (a) and tumor necrosis factor-α (TNF-α) (b) concentrations in (-●-) garlic and (-○-) placebo trials. B: represents before the 40-km cycling time trial. * Significant difference between garlic and placebo (*p* < 0.05). + Significant difference against B point in the same trial. (*p* < 0.05). Values are expressed as mean ± SE, *N* = 11.

The levels of oxidative stress and inflammation for MDA and TNF-α were significantly attenuated and accompanied by an enhanced TAC response following 4-wk garlic supplementation (Figures 3(a,b), 4(b); *p* < 0.05). Figure 3(a) shows that TAC responses were significantly higher in the garlic trial compared to those in placebo before exercise and during the exercise period (Figure 3(a), *p* < 0.05). Simultaneously, Figure 3(b) has shown that MDA levels had lower levels compared to those of placebo before exercise, 0-km, 20-km, and 40-km during the exercise challenge and after garlic supplementation (*p* < 0.05). Identically, TNF-α levels were shown to have lower levels at 10-km, and 20-km during exercise and after garlic supplementation compared to those of placebo (Figure 4(b), *p* < 0.05). There were no differences detected for the IL-6 response between the two trials (Figure 4(a), *p* > 0.05).

### 3.3. Garlic extract did not attenuate exercise-induced muscle damage markers during the exercise challenge

**Figure 5.**
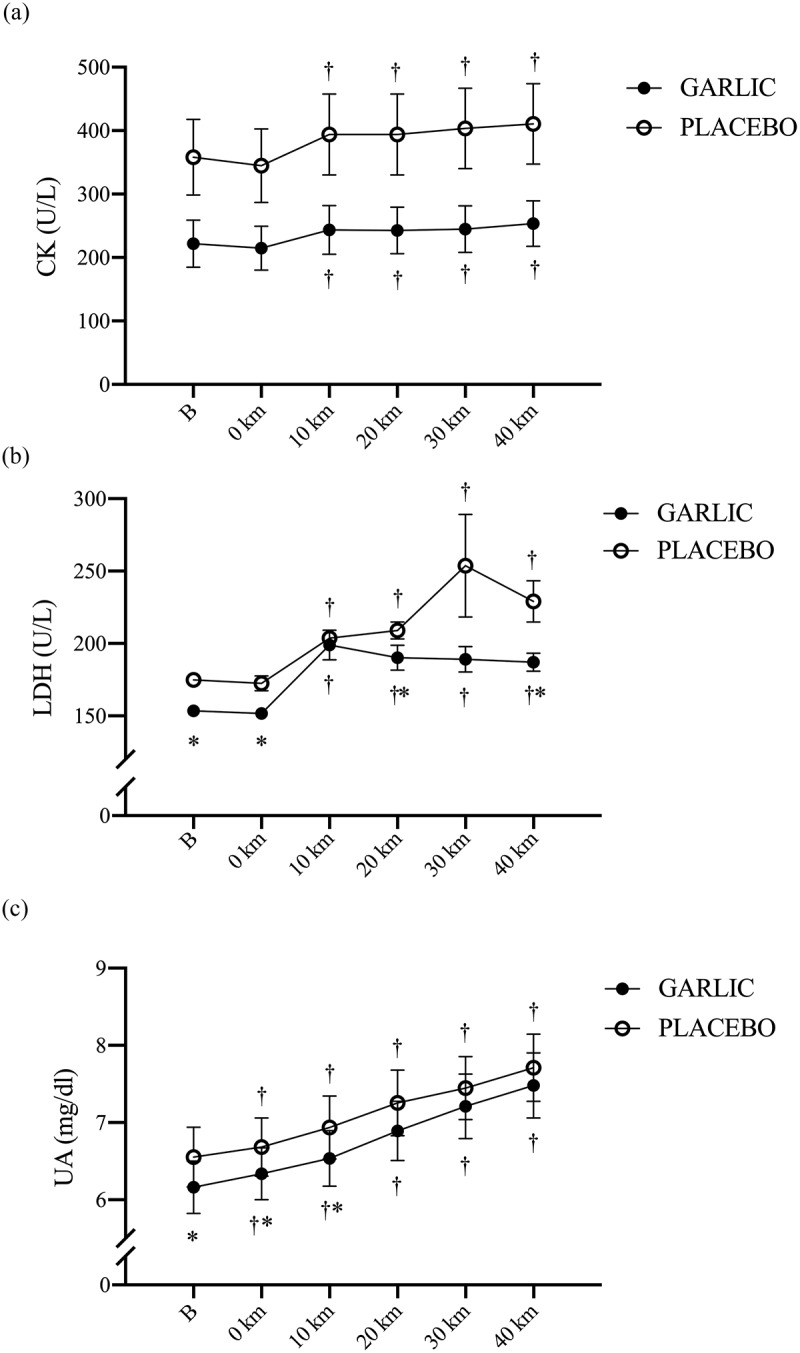
Creatine kinase (CK) (a); lactate dehydrogenase (LDH) (b), and uric acid (UA) (c) concentrations in (-●-) garlic and (-○-) placebo trials. B: represents before the 40-km cycling time trial. * Significant difference between garlic and placebo (*p* < 0.05). + Significant difference against B point in the same trial. (*p* < 0.05). Values are expressed as mean ± SE, *N* = 11.

Blood markers indicating exercise-induced muscle damage were measured after garlic and placebo supplementation. Figure 5 shows no significant difference between the two trials during the exercise challenge (Figure 5(a), *p* > 0.05). However, a higher CK response during the exercise period was found compared to that before exercise (0 km) in both trials. Interestingly, the LDH concentrations in the garlic trial were significantly lower before exercise, at 0-km, 20-km, and 40-km during exercise compared to the values for placebo (Figure 5(b), *p* < .05). In a similar manner, the protein metabolism mediator UA concentration was significantly lower before exercise, at 0-km, and 10-km than those of placebo during the exercise challenge and after oral garlic supplementation (Figure 5(c), *p* < .05).

## 4. Discussion

Both animal and human studies have shown that oral supplementation with garlic can contribute to reduced oxidative stress and inflammation. The benefits of garlic may be due to the flavonoids and sulfur compounds it contains [24,25]. In the present human study, we hypothesized that ingesting a garlic extract for 28 days would improve 40-km cycling performance. To help explain any changes in cycling performance, we investigated the physiological mechanism by which garlic supplementation affects systemic antioxidant and anti-inflammatory capacity. We also explored the effects of garlic supplementation on exercise-induced oxidative stress, inflammatory responses, and muscle damage. Our results revealed the following effects: First, performance time for a 40-km cycling challenge did not improve after oral garlic supplementation (Figure 1), and the proportion of fat utilization during exercise was not increased (Figure 2). Secondly, antioxidant enzyme TAC levels were significantly higher in the garlic group compared to placebo, while at the same time, MDA levels and exercise-induced oxidative stress were reduced (Figure 3). Thirdly, treatment with garlic significantly reduced TNF-α levels (a pro-inflammatory marker) during exercise (Figure 3(b)), whereas there was no significant difference in IL-6 levels between the two groups (Figure 3(a)). Lastly, treatment with garlic significantly decreased the LDH levels, an indicator of muscle damage, during exercise (Figure 5(b)), but there was no significant difference observed in the levels of CK and UA between the two groups. Therefore, we concluded that although garlic extract supplementation in humans for 28 days was able to significantly enhance the total antioxidant capacity (TAC), and assuage exercise-induced oxidative stress (MDA) and inflammatory response (TNF-α), the physiological adaptation caused by it was not sufficient to enhance the 40-km cycling endurance performance in subjects.

It’s well established that high levels of oxidative stress during exercise may increase muscle inflammation and damage, leading to underperformance. Thus, reducing systemic oxidative stress during exercise would be an important physiological factor in the performance of endurance exercise [26]. The present study in humans showed that garlic supplementation (1000 mg/day) for 28 consecutive days significantly increased the levels of systemic TAC (antioxidant enzymes) during exercise, and at the same time significantly reduced the levels of MDA (oxidative stress) induced by exercise (Figure 3(a,b), *p* < .05). On the other hand, in animal studies, it has been shown that treatment with garlic extract alleviated the MDA, glutathione peroxidase (GSHPx), glutathione (GSH) and glutathione disulfide (GSSG) levels [27]. Physiologically, it was suggested that the antioxidant and anti-inflammatory effects were due to SAC (S-allyl cysteine), alliin, and several fat-soluble organic sulfur-containing substances in garlic [26]. Wherein SAC apparently protected or prevented cellular damage induced by hydrogen peroxide [28]. During the average 80-minute cycling performance, supplementation with garlic significantly lowered the lipid peroxidative free radical product MDA, indicating that treatment with garlic may delay or prevent the possibility of oxidative damage in muscle cells caused by long-term energy-producing redox imbalance [29]. Nevertheless, in the present study, supplementation with garlic extract significantly delayed MDA levels induced by exercise at 4 weeks (Figure 3(b)). Moreover, Koseoglu et al. showed that garlic supplementation for 30 days (264 mg/day) significantly increased total antioxidant capacity (TAC) in 17 healthy subjects under resting conditions [30]. Based on the different experimental protocols used in the present study, 28 days of oral garlic supplementation led to significantly increased TAC (antioxidant enzyme activity) levels during the 40-km cycling challenge. Therefore, we inferred that supplementing with garlic may delay the onset of high levels of oxidative stress during exercise, thereby reducing the chance of exercise-induced inflammation and the occurrence of exercise-induced muscle damage.

The results of the present study showed that 4 weeks of garlic extract supplementation significantly delayed the inflammatory index TNF-α induced by exercise. We speculated that IL-6 is likely involved in the process (Figure 4(a,b)). Starkie et al. conducted a study in which healthy men were injected with IL-6 prior to a cycling exercise. During the 3-hour bout of cycling, the production of TNF-α was significantly inhibited by the effects of the anti-inflammatory IL-6 injection [31]. It is postulated that Ajoene (the major sulfur compound in garlic) and its sulfonyl analogs could inhibit lipopolysaccharide (LPS)-induced nitric oxide (NO)/prostaglandin E2 (PGE2) production and inducible nitric oxide synthase (iNOS)/cyclooxygenase-2 (COX-2) mRNA expression by inhibiting NF-κB and delaying the expression of pro-inflammatory cytokine TNF-α [32]. In the present human study, we observed that the levels of IL-6 increased after the 40-km cycling time trial for both the garlic and placebo groups. Thus, this indicates that the 40-km cycling challenge significantly increased the anti-inflammatory response index of IL-6 (Figure 4(a)). In addition to being an anti-inflammatory indicator, human studies have indicated that IL-6 was positively correlated to the degree of muscle glycogen consumption during exercise [33]. Pedersen showed that during exercise, muscle glycogen depletion significantly activated or induced IL-6 production [34]. The 40-km cycling challenge was indeed a high-intensity challenge for the subjects. Although the anti-inflammatory response of IL-6 after supplementation with garlic for 4 weeks was not significantly higher than with placebo, based on the results of the inflammatory index TNF-α. From this, we suggest that 4 weeks of oral garlic supplementation may delay the production of inflammatory response marker TNF-α in subjects performing high-intensity exercise. However, 28 days of garlic extract supplementation have not been reported to offer ergogenic properties during cycling time trial performance.

It has been shown that important physiological biomarkers for maintaining high-intensity endurance performance include inflammatory-related factors IL-6 and TNF-α, and muscle damage factors CK, LDH, and UA released from skeletal muscle cells [35]. Using a regime of garlic extract supplementation and a bout of intense cycling exercise, we not only observed the inflammatory-related cell factors IL-6 and TNF-α, but also examined the muscle damage indicators CK, LDH, and UA before and after the exercise challenge. It is also well recognized that increased levels of CK and LDH in the blood, cell membrane damage, and muscle damage induced by high-intensity exercise are all indicators to muscle fiber damage [36,37]. In the present human study, all subjects performed a 40-km cycling time trial at 50% VO2max intensity, and the UA and LDH concentrations after the time trial were significantly higher than before the time trial (Base-line), suggesting that our cycling protocol resulted in damage to both the cell membrane and muscle fibers (Figure 5(b,c)). However, CK, an exercise-induced muscle damage indicator, did not significantly increase. As discussed elsewhere, this finding may be explained by the fact that more time points were needed to observe the CK response in the blood [38]. Totsuka et al. (2002) performed an experimental protocol similar to the present study in untrained men, showing that blood CK levels reached a peak at 72 hours following a 90 min endurance cycling time trial (1.5 kilo pounds, 60 rpm) [39]. Unlike the blood CK levels at different time points, we observed that supplementation with garlic was not effective in delaying the increased UA levels during a 40 km cycling time trial at 50% VO2max (Figure 5(c)). In addition, Mastaloudis et al. (2006) recruited 22 runners for a 50-kilometer ultramarathon competition in which indicators of muscle damage induced by exercise was observed. They found that the muscle damage indicator LDH reached a peak (immediately) after the competition [40]. In the present study, interestingly, we observed that oral supplementation with garlic extract significantly delayed the increase in LDH during the challenge (Figure 5(b)). LDH is an important enzyme in anaerobic glycolysis and glycolysis, catalyzing the reduction and oxidation reactions between pyruvate and L-lactic acid [41]. Taken together, this suggests that supplementation with garlic extract for 4 weeks significantly delayed the onset of LDH during exercise, which appeared to be beneficial for decreasing the proportion of anaerobic glycolysis during high-intensity exercise, and significantly decreased the consumption of muscle glycogen, the main energy source during high-intensity exercise.

## 5. Conclusion

In summary, we demonstrated that 4 weeks of oral garlic supplementation (1000 mg/day) is unable to improve 40-km cycling time trial performance. Although, garlic extract enhanced total antioxidant capacity, supplementation also attenuated exercise-induced levels of lipid peroxidation MDA, inflammatory TNF-α, and muscle damage LDH during exercise. However, taking together with physiology evidence regarding cycling performance and exercise-induced energy metabolism, oxidative inflammation, and muscle damage, we suggest that supplement dosage and more muscle physiology parameters need to be verified in future studies. Consequently, the results demonstrated that 4 weeks of garlic supplementation has no ergogenic property on cycling performance in healthy adults.

## Data Availability

The data that support the findings of this study are available from the corresponding author upon reasonable request https://drive.google.com/drive/folders/1T62ImorAlRUe5qUHiTB7nKFDk8spn-nb?usp=share_link.
